# High-Sensitivity Enzymatic Glucose Sensor Based on ZnO Urchin-like Nanostructure Modified with Fe_3_O_4_ Magnetic Particles

**DOI:** 10.3390/mi12080977

**Published:** 2021-08-18

**Authors:** Qi Mao, Weixuan Jing, Weizhuo Gao, Zhengying Wei, Bian Tian, Ming Liu, Wei Ren, Zhuangde Jiang

**Affiliations:** 1 State Key Laboratory for Manufacturing Systems Engineering, International Joint Laboratory for Micro/Nano Manufacturing and Measurement Technology, Xi’an Jiaotong University, Xi’an 710049, China; mq.mq@xjtu.edu.cn (Q.M.); gaoweizhuo@stu.xjtu.edu.cn (W.G.); zywei@mail.xjtu.edu.cn (Z.W.); t.b12@mail.xjtu.edu.cn (B.T.); zdjiang@mail.xjtu.edu.cn (Z.J.); 2 Electronic Materials Research Laboratory, Key Laboratory of the Ministry of Education & International Center for Dielectric Research, State Key Laboratory for Mechanical Behavior of Materials, School of Electronic Science and Engineering, Xi’an Jiaotong University, Xi’an 710049, China; mingliu@xjtu.edu.cn (M.L.); wre@mail.xjtu.edu.cn (W.R.)

**Keywords:** enzymatic glucose sensor, Fe_3_O_4_ magnetic nanoparticles, ZnO nanoflowers, enzyme, electrochemical sensor

## Abstract

A novel and efficient enzymatic glucose sensor was fabricated based on Fe_3_O_4_ magnetic nanoparticles (Fe_3_O_4_MNPs)-modified urchin-like ZnO nanoflowers (ZnONFs). ZnONFs were hydrothermally synthesizing on a flexible PET substrate. Fe_3_O_4_MNPs were deposited on the surface of the ZnONFs by the drop-coating process. The results showed that the urchin-like ZnONFs provided strong support for enzyme adsorption. For Fe_3_O_4_MNPs, it significantly promoted the redox electron transfer from the active center of GOx to the ZnO nanoflowers beneath. More importantly, it promoted the hydrolysis of H_2_O_2_, the intermediate product of glucose catalytic reaction, and thus improved the electron yield. The sensitivity of the Nafion/GOx/Fe_3_O_4_MNPs/ZnONFs/Au/PET sensor was up to 4.52 μA·mM^−1^·cm^−2^, which was improved by 7.93 times more than the Nafion/GOx/ZnONFs/Au/PET sensors (0.57 μA·mM^−1^·cm^−2^). The detection limit and linear range were also improved. Additionally, the as-fabricated glucose sensors show strong anti-interference performance in the test environment containing organic compounds (such as urea, uric acid, and ascorbic acid) and inorganic salt (for instance, NaCl and KCl). The glucose sensor’s service life was evaluated, and it can still maintain about 80% detection performance when it was reused about 20 times. Compared with other existing sensors, the as-fabricated glucose sensor exhibits an ultrahigh sensitivity and wide detection range. In addition, the introduction of Fe_3_O_4_MNPs optimized the catalytic efficiency from the perspective of the reaction mechanism and provided potential ideas for improving the performance of other enzymatic biosensors.

## 1. Introduction

Glucose is the most widely distributed and essential monosaccharide in nature, which has significant value in many fields, such as the fermentation industry, food industry, chemical industry, or material synthesis, just to mention a few [1]. In human life and health, as an important energy source of cells, glucose has a vital impact on human health; ensuring the balance and stability of blood glucose content is essential for maintaining health. It is estimated that the number of diabetes patients in the world will reach 463 million (9.3%) in 2019, 578 million (10.2%) by 2030, and 700 million (10.9%) by 2045 [2]. Therefore, the accurate, rapid, and noninvasive detection of glucose content is an urgent problem to be solved in health care. Among the numerous methods for detecting glucose concentration, enzyme-based electrochemical glucose sensors have shown great advantages such as good selectivity, simple principle, highly sensitive performance, and strong anti-interference ability. However, due to the limitations of enzyme adsorption characteristics [3], electron yield, and electron transfer capability [4], the enzyme-based electrochemical glucose sensors can still be improved [5,6,7].

Zinc oxide (ZnO) materials have attracted extensive attention in electrochemical sensing due to their wide bandgaps (3.32 eV) and high binding energies (60 MeV) [8]. The main advantages of ZnO-based enzymatic electrochemical sensors are as follows. (1) Varieties of nanostructures such as nanoparticles nanorods, nanotubes, nanosheet, etc. have high porosity and can provide a large specific surface area, which is helpful to improve the contact area between the electrode and the liquid to be measured, and it can further improve the overall redox electron yield of the electrode, which may finally affect the catalytic sensing efficiency of the electrode. (2) The existence of numerous defects in ZnO materials caused by oxygen vacancies, zinc vacancies, and zinc interstitials lead to good surface adsorption properties. (3) As a high-quality semiconductor material, ZnO can provide an effective electron transport channel for enzyme-based electrochemical glucose sensors. Recently, many ZnO-based nanostructures have been developed and used as working electrodes of glucose sensors. Aini et al. [9] deposited ZnO nanoparticles (ZnONPs) on glassy carbon electrode to prepared a novel electrochemical glucose sensor. The results indicated that the ZnONPs provides a nano-sized environment to work as a direct electron transmission channel which can accelerate the electron transfer. Fan et al. [10] fabricated enzyme glucose sensors using ZnO nanotubes by the selective dissolution of ZnO nanorods, and they investigated the influence of surface quality parameters of ZnO nanotubes arrays on the performance of glucose sensors. It is shown that the ZnO nanotubes-based glucose sensor can provide a higher specific surface area and obtain a better understanding than that of the ZnO nanorods type. However, the morphology of ZnO nanorods, nanotubes, and nanoplates has a dense structure arrangement, nanowires and nanotubes are closely connected, and the porosity of the structure space is small [11,12]. In contrast, ZnO nanoflowers (ZnONFs) have a natural three-dimensional structure that can construct continuous micro-networks. Based on this characteristic, the large specific surface area provided by ZnONFs can be more stable [13]. Based on using ZnO nanostructures to improve the electronic transport performance and enzyme adsorption performance of enzymatic electrochemical sensors, the deposition of different kinds of nanoparticles on ZnO nanostructures to improve the redox electron transfer rate has become a research hotspot in recent years. Metal materials such as gold [14], silver [15], platinum [16], and metal oxides materials such as NiO [17], CuO [18], and graphene [19] based materials have been introduced to prepare enzyme glucose sensors. However, the above research’s core is to use the excellent conductivity of metals, metal oxides, and graphene materials to improve electronic transmission efficiency. To further improve the sensor’s performance, it is urgent to explore the possibility of enhancing the glucose sensor’s performance from the perspective of the catalytic enzyme mechanism [4].

As a magnetic material, ferrosoferric oxide (Fe_3_O_4_) has attracted much attention in the fields of color imaging [20], bionanotechnology [21], medical diagnosis [22], controlled drug delivery [23], and so on. Fe_3_O_4_ contained both Fe^3+^ and Fe^2+^, which are disorderly arranged in the octahedral position. The electrons can transfer rapidly between the two oxidation states of iron, making Fe_3_O_4_ have excellent conductivity [24]. In addition, Fe_3_O_4_ magnetic nanoparticles (Fe_3_O_4_MNPs) have a special inverse spinel crystal structure, which can accelerate the electron transfer in H_2_O_2_, thus promoting the hydrolysis of H_2_O_2_ to produce H_2_O and O_2_ [25]. This property is also known as the peroxidase-like activity of ferromagnetic nanoparticles. Enzyme-modified Fe_3_O_4_MNPs have been proved to combine magnetic properties’ separating power with the enzyme conjugate [26]. Based on the above material properties, the modification of Fe_3_O_4_MNPs with specific enzymes may improve enzyme sensors’ performance to a certain extent.

Among the numerous methods for detecting glucose concentration, enzyme-based electrochemical glucose sensors have shown great advantages such as good selectivity, highly sensitive performance, and strong anti-interference ability. In this paper, ZnONFs were synthesized by the hydrothermal method and modified with Fe_3_O_4_MNPs to construct a high-performance enzyme glucose sensor. The peroxidase-like activity of the Fe_3_O_4_MNPs and the ZnO nanoflowers’ three-dimensional structure were combined to produce a synergistic effect. On the one hand, it provides the necessary conduction channel for the electrons on the electrode surface. On the other hand, it promotes the catalytic reaction of enzymes and improves the electron yield. This methodology is simple and effective. By optimizing the electrode’s micro nanostructure and introducing magnetic nanoparticles to improve the enzyme reaction principle, the enzyme glucose sensor’s performance was improved. It provides a new idea for the preparation of a high-performance enzymatic glucose sensor.

## 2. Experimental

### 2.1. Materials and Chemicals

The substrate material used in the experiment is flexible PET (Resistance 5–7 Ω/sq) which was purchased from Guangzhou Tiansheng Technology Co., Ltd (Guangzhou, China). Hexamethylenetetramine (99.0 wt %), zinc nitrate hexahydrate (99.9 wt %), zinc acetate dihydrate (99.9 wt %), and iron oxide (Fe_3_O_4_, 98 wt % metals basis, ≤30 nm) were provided by Aladdin Reagent Co., Ltd (Shanghai, China). Uric acid (UA, ≥99.0 wt %), urea (U, 99.0–100.5 wt %), sodium chloride (NaCl, ≥99.5%), ascorbic acid (AA, >99.0 wt %), nafion (5 wt %), glucose oxidase (GOx, 200 U·mg^−1^), D-(+)-glucose (99.5 wt%), and phosphate buffer solution (PBS, 10 mM, pH 7.4) were prepared with Na_2_HPO_4_·12H_2_O and KH_2_PO_4_ mixed in deionized water and potassium chloride (KCl, ≥99%), and purchased from Sigma-Aldrich (Burlington, MA, USA). Absolute ethyl alcohol was provided by Tianjin ZhiYuan Chemical Reagent Factory Co., Ltd (Tianjin, China). All the deionized water used in the experiment provided by an ultra-clean laboratory. The glucose solution (0.25 M) was prepared by glucose and PBS solution and held at least 24 h after preparation for mutarotation.

### 2.2. Apparatus and Software

The nanostructure of the Fe_3_O_4_MNPs and ZnONFs thin film was observed by Field-Emission Scanning Electron Microscope (FESEM, GeminiSEM 500, Zeiss, Oberkochen, Germany). The crystal structures of Fe_3_O_4_MNPs and ZnONFs thin film were measured by X-ray diffraction (XRD). The magnetic properties of Fe_3_O_4_ nanoparticles were measured by vibrating sample magnetometer (VSM-7410, Lake Shore Cryotronics, Westerville, OH, USA), and the magnetic field range was set off -3kOe to 3kOe. The electrochemical tests such as electrochemical impedance spectroscopy, cyclic voltammogram characterization, and amperometric response were conducted on an electrochemical workstation (CHI660D, CH Instruments, Austin, TX, USA).

### 2.3. Preparation of the Fe_3_O_4_MNPs Modified ZnONFs Electrodes

**Figure 1:**
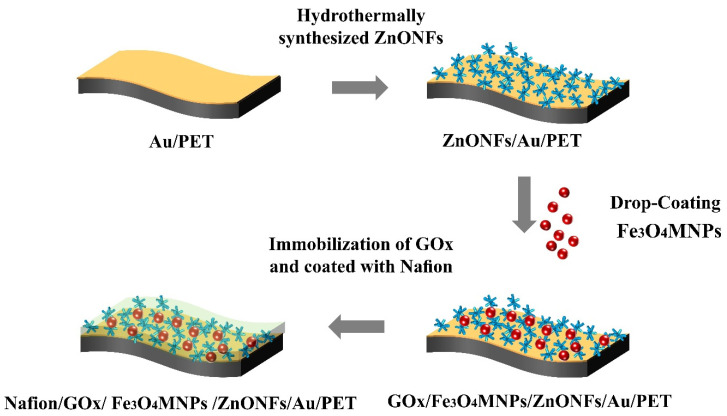
The fabrication process of the Nafion/GOx/Fe_3_O_4_MNPs/ZnONFs/Au/PET electrode.

As shown in Figure 1, there are four procedures to prepare the Fe_3_O_4_NPs modified ZnONFs enzymatic glucose sensors. Firstly, the flexible PET was deposited with Au film to enhance the conductivity of the basic substrates. Secondly, ZnONFs were synthesized by the hydrothermal method on the prepared substrates. Then, Fe_3_O_4_MNPs were modified on ZnONFs by the drop-coating method to obtain the Fe_3_O_4_MNPs/ZnONFs/Au/PET nanostructure. Finally, GOx was adsorbed on Fe_3_O_4_MNPs/ZnONFs/Au/PET substrates and coated with Nafion to cover it for fixation. The electrochemical analysis was based on the traditional three-electrode systems. The working electrode, auxiliary electrode, and reference electrode used in the experiment are as-fabricated electrodes, platinum wire, and Ag/AgCl.

#### 2.3.1. Pretreatment of the PET Substrates

Firstly, the PET substrate was cleaned with ethanol solution to remove impurities; then, the residual ethanol solution was flushed by DI water. The prepared substrates are placed in a super clean room at a temperature of 22 °C until the surface is completely dried. Then, Au film of thickness 50 nm was deposited on PET substrates by ion sputter to enhance the flexible substrates’ conductivity. Finally, the Au/PET was cut into a rectangle of 0.5 cm × 2 cm as the substrates of the enzyme sensor’s working electrodes.

#### 2.3.2. Hydrothermal Preparation of ZnONFs

ZnONFs were synthesized using the hydrothermal method. Firstly, 0.35 g of hexamethylenetetramine and 0.742 g of zinc nitrate hexahydrate were dissolved into 100 mL of deionized water, and the concentration of Zn^2+^ was 25 mM. Secondly, the solution’s temperature from room temperature is raised to 90 °C and is kept stirring until it is completely mixed. Finally, the Au/PET substrates were quickly put into the growth solution and placed in a 90 °C water bath for 2.5 h to prepare ZnONFs. Thus, the ZnONFs structure was firmly grown on the Au/PET substrates so it forms the ZnONFs/Au/PET substrates.

#### 2.3.3. Drop-Cast Coating Fe_3_O_4_MNPs on the ZnONFs/Au/PET Substrates

Fe_3_O_4_MNPs were electrostatically adsorbed on ZnONFs by a drop-coating method. First, 2.5 g of iron oxide were dissolved into 50 mL of deionized water. Then, the mixed liquids were dispersed by ultrasonic bath for 15 min to configured as Fe_3_O_4_ suspension. The prepared suspension was stored at 4 °C until being used, and the content of Fe_3_O_4_MNPs in the suspension was about 50 mg/mL. Finally, applied 10 µL of the prepared suspension on the surface of ZnONFs/Au/PET substrates and dried in the ultra-clean laboratory. Thus, the Fe_3_O_4_MNPs/ZnONFs/Au/PET substrates were achieved.

#### 2.3.4. Drop-Coated GOx on the As-Fabricated Electrodes

GOx solution (40 mg·mL^−1^) was prepared to modify the enzyme catalytic electrode. Subsequently, 10 μL of fresh GOx solution was dropped on the surface of the as-fabricated substrates and then dried naturally at 4 °C in the air to achieve the GOx/Fe_3_O_4_MNPs/ZnONFs/Au/PET and GOx/ZnONFs/Au/PET substrates. After that, 5 μL Nafion solution was coated on the as fabricated substrates to form an ion transparent membrane on top. Thus, the Nafion/GOx/Fe_3_O_4_MNPs/ZnONFs/Au/PET and Nafion/GOx/ZnONFs/Au/PET enzymatic electrodes were achieved. The prepared electrode is related to the lead, fixed with silver conductive adhesive, and used as the working electrode to form a three-electrode system together with the reference electrode and counter electrode, to carry out further electrochemical tests. To maintain the biological activity of GOx, these as-fabricated electrodes should be stored at 4 °C.

### 2.4. Measurement of Electrochemical Properties of the As-Fabricated Electrodes

The electrochemical test is carried out by using the traditional three electrode system, in which the working electrode is the as-fabricated electrode in this paper, platinum wire is used as the auxiliary electrode, and Ag/AgCl is used as the reference electrode. An aerometric i-t curve was used to characterize the as-fabricated electrode’s performance. In the measurement process, it is necessary to ensure the stability of the background current to achieve an accurate current response. Then, 100 μL glucose solution was added into 50 mL of PBS solution obtain the current response curve. After waiting for a stable response current, we repeated the above steps until the current response curve was saturated. Elec-trochemical impedance spectroscopy (EIS) was used to characterize the electron transfer ability of the electrodes. The electrochemical impedance spectroscopy of the as-fabricated glucose sensors was tested in PBS solution. The frequency is from 0.01 Hz to 100 kHz, and the bias potential is +0.1 V. The redox process was described by cyclic voltammetry. A cyclic voltammogram was performed in a 3 mM glucose solution with a scan rate of 50 mV s^−1^ and potential range of −0.8 V to +0.8 V. The sensitivity of the as-fabricated glucose sensor to urea, uric acid, ascorbic acid, sodium chloride, and potassium chloride was also evaluated. All the above electrochemical tests were carried out at room temperature.

## 3. Results and Discussion

### 3.1. The Morphology and Composition of the Fe_3_O_4_MNPs/ZnONFs/Au/PET and ZnONFs/Au/PET Substrate

**Figure 2:**
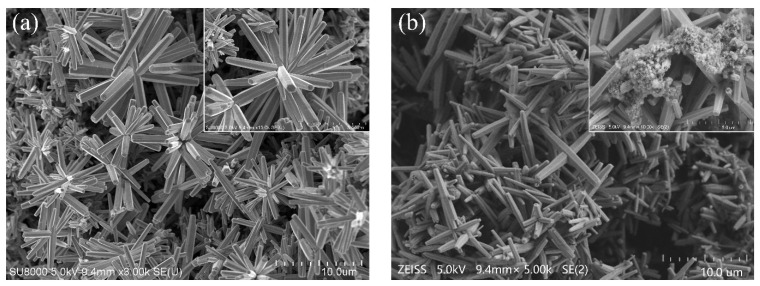
FE-SEM micrographs of (**a**) ZnONFs/Au/PET and (**b**) Fe_3_O_4_MNPs/ZnONFs/Au/PET substrates.

The morphology of the ZnONFs/Au/PET substrate is shown in Figure 2a. Each urchin-like ZnONF is constructed with tens of ZnO nanorods, which are radially clustered together. The ZnO nanorods have a typical hexagonal wurtzite crystal structure, which suggests that the ZnONFs are well-ordered and highly crystallized. The diameter of each ZnO nanorod is about 200 nm. Moreover, the average size of ZnONFs shows good consistency, which was about 3 μm. It is worth knowing that different ZnONFs cluster and stack together to form a three-dimensional porous structure. Urchin-like spines support the ZnONFs in the same plane to form plenty of polymorphic voids. At the same time, the disordered stacking of ZnONFs forms multipath holes in the longitudinal direction. In order to comprehend the detail of Fe_3_O_4_MNPs deposited on ZnONFs, the image of the Fe_3_O_4_MNPs/ZnONFs/Au/PET substrate is depicted in Figure 2b. It can be seen from the image that the structural morphology of ZnONFs in the Fe_3_O_4_MNPs/ZnONFs/Au/PET substrate is consistent with Figure 2a. In particular, Fe_3_O_4_MNPs are stably deposited in the void of a three-dimensional ZnONFs structure. For one thing, the ZnONFs provide more stable support for Fe_3_O_4_MNPs; for another, Fe_3_O_4_MNPs are mainly attached to the surface of the ZnONFs structure, which lays a good contact condition for subsequent electrochemical catalytic sensing.

**Figure 3:**
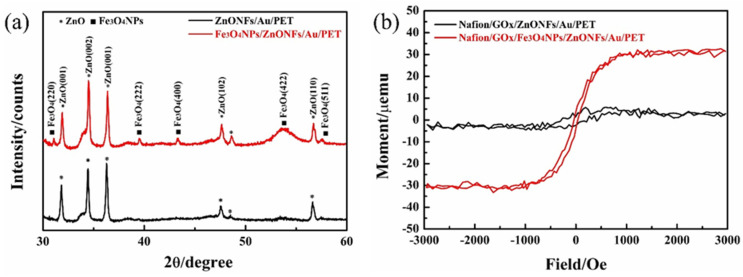
(**a**) XRD curves of ZnONFs/Au/PET and Fe_3_O_4_MNPs/ZnONFs/Au/PET substrates. (**b**) Magnetic hysteresis loops behavior of ZnONFs/Au/PET and Fe_3_O_4_MNPs/ZnONFs/Au/PET.

The XRD patterns of the ZnONFs and Fe_3_O_4_MNPs/ZnONFs substrate are illustrated in Figure 3a. The black curve indicates that the XRD spectrums of the ZnONFs thin-film structure show evident and intense diffraction peaks of (001), (002), (102), and (110) planes, which corresponded to the crystal structure of ZnO (JCPDS cards #36-1451). The close-packed hexagonal structure and high crystallinity of ZnO are confirmed. In addition to the ZnO X-ray diffraction peaks, the Fe_3_O_4_MNPs/ZnONFs thin-film structure exhibited the characteristic diffraction peaks at 30.0°, 43.0°, 53.4°, and 56.9°, which corresponded to the (220), (400), (422), and (511) planes, respectively (shown in the red curve), which confirms the monoclinic crystal structure of Fe_3_O_4_ lattice (#75-1610). In addition, combined with the characterization data of the X-ray curve, the average crystalline size of Fe_3_O_4_MNPs can be calculated by the Debye–Schell formula shown in Equation (1).

Dhkl = k (λ/β cosθ)(1)

The grain diameter perpendicular to the crystal plane is presented as Dhkl, and the Scherrer constant is k (usually 0.89). The incident wavelength of the X-ray is λ (usually 0.15418 nm), the Bragg diffraction angle is θ, and the half-maximum width of the diffraction peak is β. It is found that the average particle size of the Fe_3_O_4_MNPs is about 33.1 nm. The above analysis shows that both of the ZnONFs/Au/PET and Fe_3_O_4_MNPs/ZnONFs/Au/PET substrates have a well-crystallized ZnO nanostructure. The Fe_3_O_4_MNPs/ZnONFs thin film also shows a typical high crystalline cubic spinel structure of Fe_3_O_4_ with an average size of about 33.1 nm. All in all, X-ray diffraction confirmed the formation of the Fe_3_O_4_MNPs/ZnONFs composite film structure.

The magnetic field dependence of magnetization of the ZnONFs/Au/PET and Fe_3_O_4_MNPs/ZnONFs/Au/PET substrates are presented in Figure 3b. The ZnONFs/Au/PET substrate curve is basically a horizontal line that indicates that ZnONFs thin film is diamagnetic. In contrast, the hysteresis curve of Fe_3_O_4_MNPs/ZnONFs/Au/PET substrates shows a typical S-shaped hysteresis loop, which means the Fe_3_O_4_MNPs/ZnONFs thin film has actual superparamagnetic behavior in shallow coercivity fields. The saturation magnetization value is about 30 µemu/g. The results show that Fe_3_O_4_MNPs were reliably deposited on ZnONFs, and the Fe_3_O_4_MNPs/ZnONFs thin film has certain superparamagnetism. These results are consistent with the SEM pictures in Figure 2 and XRD curves in Figure 3a.

The magnetic field dependence of magnetization of the ZnONFs/Au/PET and Fe_3_O_4_MNPs/ZnONFs/Au/PET substrates are presented in Figure 3b. The ZnONFs/Au/PET substrate curve is basically a horizontal line that indicates that ZnONFs thin film is diamagnetic. In contrast, the hysteresis curve of Fe_3_O_4_MNPs/ZnONFs/Au/PET substrates shows a typical S-shaped hysteresis loop, which means the Fe_3_O_4_MNPs/ZnONFs thin film has actual superparamagnetic behavior in shallow coercivity fields. The saturation magnetization value is about 30 µemu/g. The results show that Fe_3_O_4_MNPs were reliably deposited on ZnONFs, and the Fe_3_O_4_MNPs/ZnONFs thin film has certain superparamagnetism. These results are consistent with the SEM pictures in Figure 2 and XRD curves in Figure 3a.

### 3.2. Electrochemical Characterization of the Nafion/GOx/ZnONFs/Au/PET and Nafion/GOx/Fe_3_O_4_MNPs/ZnONFs/Au/PET Glucose Sensors

#### 3.2.1. Characterization of EIS Curve

**Figure 4:**
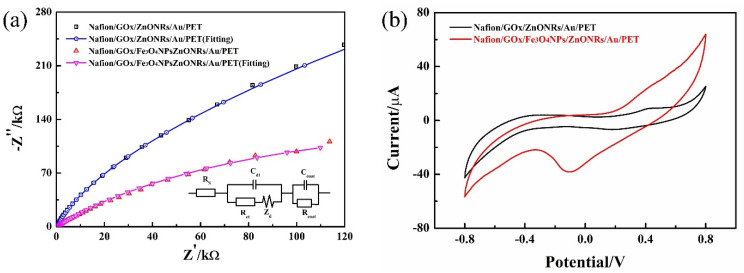
(**a**) The EIS curve of the Nafion/GOx/ZnONFs/Au/PET and Nafion/GOx/Fe_3_O_4_MNPs/ZnONFs/Au/PET electrodes in PBS solution. The inset shows the equivalent circuit diagram of the sensors. The frequency is from 0.01 Hz to 100 kHz, and the bias potential is +0.1 V. (**b**) Cyclic voltammograms in 3 mM glucose solution on the ZnONFs/Au/PET electrode (black line) and Fe_3_O_4_MNPs/ZnONFs/Au/PET electrode (red line). The fitting curves of the ZnONFs/Au/PET electrode (blue line) and Fe_3_O_4_MNPs/ZnONFs/Au/PET electrode (magenta line).

The Nyquist diagrams of the Nafion/GOx/ZnONFs/Au/PET and Nafion/GOx/Fe_3_O_4_MNPs/ZnONFs/Au/PET glucose sensors are shown in Figure 4a. The high-frequency characteristic of the Nyquist curve describes the electron migration characteristic. The calculation shows that the R_ct_ of the ZnONFs/Au/PET electrodes is about 463.1 ± 9.05 kΩ (black curve), while the R_ct_ of the Fe_3_O_4_MNPs/ZnONFs/Au/PET electrodes is about 366.1 ± 10.67 kΩ (red curve). The electron transfer resistance shows a decreasing trend, and this reveals that Fe_3_O_4_MNPs can promote electron transfer in the electrode process to a certain extent.

The electrical equivalent circuit is fitted by ZSimpWin software, which is consistent with the actual measurement results, as shown in Figure 4a as an inset picture. On a planar electrode, the electrode reaction process can be regarded as a process controlled by both electron transfer and diffusion. R_s_ represents the electrolyte solution resistance, R_ct_ represents the charge transfer resistance, C_dl_ represents the double layer capacitance, Z_d_ represents the Warburg impedance, and R_coat_ and C_coat_ are the Warburg diffusion elements corresponding respectively to the diffusion through the Nafion membrane. The electrochemical impedance spectroscopy of the as-fabricated glucose sensors was tested in PBS solution. The frequency is from 0.01 Hz to 100 kHz, and the bias potential is +0.1 V.

#### 3.2.2. Cyclic Voltammogram Characterization

The Cyclic voltammograms in Figure 4b shows that both oxidation and reduction peaks can be detected at the as-fabricated electrodes. It is worth noting that the redox current of the Fe_3_O_4_MNPs modified working electrode was significantly higher than that of ZnONFs working electrode, which indicated that the Fe_3_O_4_MNPs play a positive role in improving the enzyme catalytic reaction and the electrochemical performance of the working electrode.

**Figure 5:**
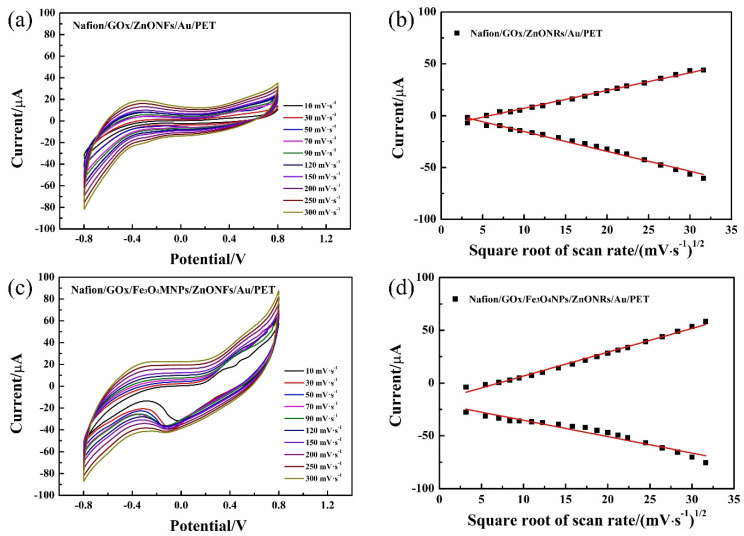
(**a**) The CV curve of the ZnONFs/Au/PET electrode. (**b**) Linear fitting curves of redox current and the square root of scanning rate of glucose ZnONFs/Au/PET electrode. (**c**) Fe_3_O_4_MNPs modified electrode in 3 mM glucose solution. (**d**) Linear fitting curves of redox current and the square root of scanning rate of Fe_3_O_4_MNPs modified electrode.

Cyclic voltammograms evaluated the electrokinetics that occurred in the glucose detection system. The changing trend of redox current was characterized by a 3 mM glucose solution concentration under the scanning rate of 10 to 1000 mV·s^−1^. Figure 5a,c indicated that both Nafion/GOx/ZnONFs/Au/PET and Nafion/GOx/Fe_3_O_4_MNPs/ZnONFs/Au/PET electrodes have obvious oxidation and reduction peaks, and the oxidation–reduction peak increases gradually with the increase of scanning rate, respectively. Furthermore, the peak currents of both I_pa_ and I_pc_ exhibit a linear relationship with the square root of the scan rate, as shown in Figure 5b,d, which means that both enzymatic electrodes were diffusion-controlled processes.

#### 3.2.3. Amperometric Response

**Figure 6:**
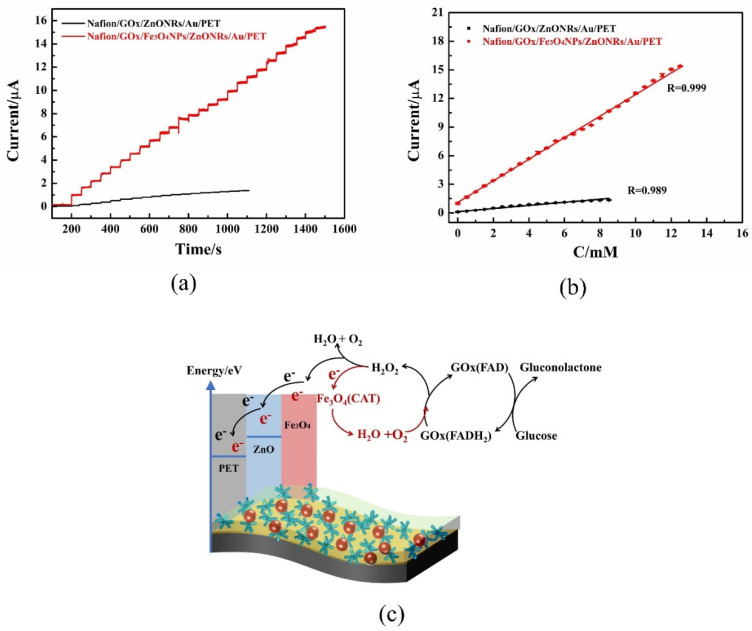
(**a**) The amperometric response of the as-fabricated glucose sensors for the successive addition of 100 μL of glucose solution in 50 mL of PBS at an applied bias potential of +0.8 V. (**b**) The corresponding calibration plots of the as-fabricated glucose sensors. (**c**) The redox reaction mechanism and electron transport scheme on the as-fabricated enzymatic working electrode.

Figure 6a show that both as-fabricated electrodes are with proportionate and stable electrocatalytic activities. The amperometric response of the Nafion/GOx/Fe_3_O_4_MNPs/ZnONFs/Au/PET electrode was much higher than that of the Nafion/GOx/ZnONFs/Au/PET electrode. This indicates that the Nafion/GOx/Fe_3_O_4_MNPs/ZnONFs/Au/PET electrode is more sensitive to the concentration of glucose in this test environment. Figure 6b indicated that there is a linear relationship between the response current and the concentration of glucose solution, and the correlation coefficient is as high as R = 0.999. Figure 6c shows the redox reaction mechanism and electron transport scheme of the glucose sensing. The introduction of Fe_3_O_4_MNPs provides more enzyme adsorption sites on the surface of ZnONFs nanomaterials, which play a catalytic role from two aspects.

On the one hand, as discussed in Section 3.2.1, the introduction of Fe_3_O_4_MNPs improves the electron transfer efficiency between the solution and the electrode surface and also promotes electron transfer in the reaction process. On the other hand, as shown in Equations (3) and (4), the intrinsic peroxidase-like activity of Fe_3_O_4_MNPs [25] can promote the hydrolysis of the intermediate product H_2_O_2_, which can form H_2_O and O_2_, thus generating more electrons and promoting the electron yield. More importantly, the enzyme catalytic process consumes O_2_, and the O_2_ produced by hydrogen peroxide hydrolysis supplements the O_2_ content in the solution, which promotes the catalytic cycle of the enzyme and accelerates the electronic production. Based on the combination of these two aspects, the introduction of Fe_3_O_4_MNPs can significantly improve the enzyme-catalyzed glucose sensor’s performance.

(2) $$\mathrm{Glucose}+{\mathrm{GO}}_{x}\left(\mathrm{FAD}\right)\overset{\;}{\to }\mathrm{Gluconic}\;\mathrm{acid}+{\mathrm{GO}}_{x}\left({\mathrm{FADH}}_{2}\right)$$

(3) $${\mathrm{GO}}_{x}\left({\mathrm{FADH}}_{2}\right)+O_{2}\overset{\;}{\to }{\mathrm{GO}}_{x}\left(\mathrm{FAD}\right)+H_{2}O_{2}$$

(4) $$2H_{2}O_{2}\;\overset{{\mathrm{Fe}}_{3}O_{4}}{\to }\;2H_{2}O+{\;O}_{2}+2e-$$

The sensitivities of the Nafion/GOx/ZnONFs/Au/PET sensor are 0.57 μA·mM^−1^·cm^−2^, and the Nafion/GOx/Fe_3_O_4_MNPs/ZnONFs/Au/PET is 4.52 μA·mM^−1^·cm^−2^, which is 7.93 times higher than the other. The low detection limits are 0.105 μM and 0.089 μM, respectively. The Nafion/GOx/ZnONFs/Au/PET sensors’ linear range reached 8.5 mM, and the Nafion/GOx/Fe_3_O_4_MNPs/ZnONFs/Au/PET sensor extended to 12.5 mM. Human blood glucose is maintained at a constant level of 4 to 6.5 mM, and the linear range of blood glucose monitoring should cover the range of 3–8 mM. Both as-fabricated sensors meet the requirements of human glucose monitoring. The results show that the introduction of Fe_3_O_4_ magnetic nanoparticles can improve glucose sensor performance based on ZnONFs nanostructure. The performance comparison between this work and other similar literature is shown in Table 1. From the current research on the enzymatic glucose sensor, it can be seen that the main methods to improve the performance of the sensor focus on two aspects: one is to prepare nano mechanism materials with high specific surface areas, such as CNT-Mucin and ZnO nanostructure, to increase the solid–liquid contact area of an enzymatic glucose sensor, improving the electron yield of the enzyme-catalyzed reaction, and then leading to the improvement of performance. In this aspect, the ZnONFs structure prepared in this work can provide 3D morphology support, which has the same advantages as other literature. Another option is to introduces other superconducting materials, such as graphene and Pt, to improve the efficiency of electron transfer, thereby improving the performance of the sensor. Compared with the sensor of another material system, the Fe_3_O_4_NPs-modified sensor has better sensitivities. These are mainly due to the Fe_3_O_4_MNPs having a special peroxidase-like activity, which can promote the hydrolysis of H_2_O_2_ to produce H_2_O and O_2_ and enhance the electron yield of the enzyme catalytic process. In conclusion, to compare with other similar sensors, the as-fabricated Nafion/GOx/Fe_3_O_4_NPs/ZnONFs/Au/PET sensors also reach the equivalent or even better sensitivity.

#### 3.2.4. The Anti-Interference Capability of the As-Fabricated Glucose Sensors

Considering that the glucose sensor may be used in a complex environment that contained various substances, the prepared sensor should have a certain anti-interference performance to ensure detection accuracy. It is necessary to verify the current response of the glucose sensors to other potential substances. The following procedure is performed to verify the anti-interference of the as-fabricated glucose sensor.

**Figure 7:**
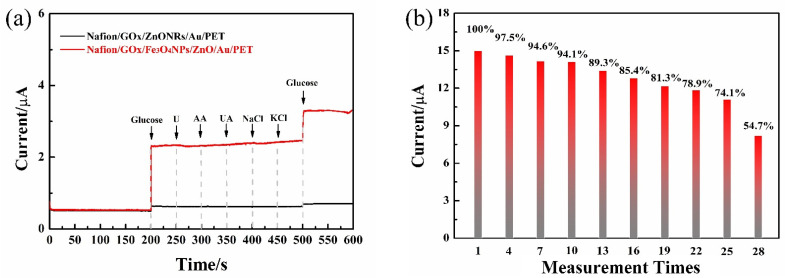
(**a**) The anti-interference of the as-fabricated glucose sensors. (**b**) The service life of Nafion/GOx/Fe_3_O_4_MNPs/ZnONFs/Au/PET glucose sensors.

Glucose (3 mM), urea (U, 0.5 mM), uric acid (UA, 0.5 mM), ascorbic acid (AA, 0.1 mM), NaCl (0.5 mM), KCl (0.5 mM), and glucose (3 mM) were added in 50 mL PBS solution in turns at the experimental environment. The amperometric current was observed to obtain the ability of the as-fabricated sensors to respond to these potential substances. As shown in Figure 7a, when glucose solution was added, the oxidation currents response is obvious, and a step is formed. When U, AA, UA, NaCl, and KCl were added to PBS solution successively, the oxidation currents of the two glucose sensors were almost unchanged, and a step was formed after glucose was added later. It suggests that the Nafion/GOx/ZnONFs/Au/PET and Nafion/GOx/Fe_3_O_4_MNPs/ZnONFs/Au/PET glucose sensors are not easy to be interfered with by other substances and have high anti-interference performance for glucose detection. The following two contributions may provide a basis: the advantage of enzyme catalytic sensing is the specific reaction of glucose oxidase to glucose, and the Nafion is coated to provide a membrane to interfere with anions such as UA and AA, respectively.

#### 3.2.5. The Service Life of the Glucose Sensors

Figure 7b indicates the Nafion/GOx/Fe_3_O_4_MNPs/ZnONFs/Au/PET glucose sensors’ service life. The glucose sensors were kept in a 3 mM glucose solution. The device’s service life was evaluated by recording the current intensity in the response’s cyclic voltammogram. The abscissa is the measurement times of the same sensor, and the ordinate is the attenuation of the sensor’s current response after different measurements. It is shown that Nafion/GOx/Fe_3_O_4_MNPs/ZnONFs/Au/PET glucose sensors retained 85.4% of the initial oxidation current after being reused 16 times, respectively. After being reused 19 times, the glucose sensors’ oxidation currents decreased to 81.3% of the initial values. This suggests that the sensor’s measurement accuracy is maintained at about 80% of the initial state when it is reused about 20 times. When repeatedly used 28 times, the Nafion/GOx/Fe_3_O_4_MNPs/ZnONFs/Au/PET glucose sensors’ performance rapidly decayed to 54.7% of the initial values, indicating that the sensor reached the limit of its service life. For the enzyme sensor, the decline of its performance is mainly due to the loss of enzyme and the attenuation of enzyme activity. The covering Nafion ion membrane is an effective way to prevent excessive loss of enzymes. In addition, it should be noted that storing the sensor in a suitable environment can also effectively ensure enzyme activity.

## 4. Conclusions

This study presents a simple approach to fabricate an efficient enzymatic glucose sensor based on urchin-like ZnO nanoflowers modified with Fe_3_O_4_ MNPs. The excellent conductivity of Fe_3_O_4_MNPs can promote the transfer of redox electrons from a GOx active center to ZnONFs. As nanoscale magnetic particles, the intrinsic peroxidase-like activity of Fe_3_O_4_MNPs can promote the hydrolysis of H_2_O_2_, the intermediate product, and generate more electrons to promote the electron yield. The sensitivity of the Nafion/Fe_3_O_4_MNPs/ZnONFs/Au/PET glucose sensor increased to 4.52 μA·mM^−1^·cm^−2^, and the detection limit reached 0.089 μM. The linear range was 0.089–12.5 mM, which could match the requirement of clinical physiological blood glucose detection in the range of 3–8 mM physiological blood glucose level. The as-fabricated glucose sensors exhibited excellent selectivity in a test environment containing organic compounds and inorganic salt. The evaluation of the glucose sensors’ service life demonstrated that the device’s current response maintained 78.9% of its initial value after reuse 22 times. The results can improve the electrochemical biosensors’ performance based on the combination of magnetic nanoparticles and nanostructured matrix materials.

## Figures and Tables

**Table 1:** Comparison of the performance of the enzyme glucose sensors.

Electrodes	Sensitivity μA·mM^−1^·cm^−^^2^	Detection Limits μM	Linear Range mM	Michaelis–Menten Constant mM	Reference
Nafion/GOx/ZnONFs/Au/PET	0.57	0.105	0.15 × 10^−3^–8.5	4.48	This work
Nafion/GOx/Fe_3_O_4_NPs/ZnONFs/Au/PET	4.52	0.089	0.089 × 10^−3^–12.5	14.65	This work
Nafion/GOx/ZnO/rGO/ITO	2.29	1.0	0–6.5	10.79	[27]
Nafion/GOx/Au-ZnO/rGO/ITO	5.21	0.5	0–11.0	8.15	[10]
GOx/CNT-Mucin/Pt	15 mA·mM^−1^·cm^−2^	100	2 μM–3.2 mM	Not reported	[28]
CdS/ITO with GOx mixed electrolyte	1.345	0.1	2–225	Not reported	[29]
GOx/ZnO/C/Paper	8.24	59.5	0–5.0	Not reported	[30]
GOx/SiO_2_/Lig/CPE	0.78	145	0.5–9.0	Not reported	[31]

Abbreviations: Au/PET: gold doped on PET; CNT-Mucin: carbon nanotubes–mucin composite; AuNPs: gold nanoparticles; SiO_2_/Lig: silica doped lignin; CPE: carbon paste electrode.
